# One bout of endurance exercise does not change gene expression or proliferation in a C26 colon carcinoma in immunocompetent mice

**DOI:** 10.1007/s00432-023-05447-x

**Published:** 2023-10-16

**Authors:** Nik Mahnic, Alessia Geremia, Tobias Straub, Sabrina Zorzato, Martin Schönfelder, Irene von Lüttichau, Katja Steiger, Maximilian Michael Saller, Bert Blaauw, Henning Wackerhage

**Affiliations:** 1 Professorship of Exercise Biology, School of Medicine and Health, Technical University of Munich, Munich, Germany; 2 Venetian Institute of Molecular Medicine (VIMM), Padua, Italy; 3 Department of Biomedical Sciences, University of Padova, Padua, Italy; 4 Bioinformatics Core, Biomedical Center, Faculty of Medicine, Ludwig-Maximilians-University (LMU), Munich, Germany; 5 Kinderklinik München Schwabing, Department of Pediatrics and Children’s Cancer Research Center, TUM School of Medicine and Health, Technical University of Munich, Munich, Germany; 6 Comparative Experimental Pathology, Institute of Pathology, School of Medicine and Health, Technical University of Munich, Munich, Germany; 7 Department of Orthopaedics and Trauma Surgery, Musculoskeletal UniversityCenter Munich (MUM), Ludwig-Maximilians-University (LMU) University Hospital, LMU Munich, Fraunhoferstraße 20, 82152 Planegg-Martinsried, Germany

**Keywords:** Cancer, Exercise, C26 colon carcinoma, BALB/c mice

## Abstract

**Purpose:**

Exercise typically reduces tumour growth, proliferation and improves outcomes. Many of these effects require exercise to change gene expression within a tumour, but whether exercise  actually affects gene expression within a tumour has not been investigated yet. The aim of this study was, therefore, to find out whether one bout of endurance exercise alters gene expression and proliferation in a C26 carcinoma in immunocompetent mice.

**Methods:**

BALB/c were injected with C26 colon carcinoma cells. Once the tumours had formed, the mice either ran for 65 min with increasing intensity or rested before the tumour was dissected. The tumours were then analysed by RNA-Seq and stained for the proliferation marker KI67.

**Results:**

One bout of running for 65 min did not systematically change gene expression in C26 carcinomas of BALB/c mice when compared to BALB/c mice that were rested. However, when analysed for sex, the expression of 17, mostly skeletal muscle-related genes was higher in the samples of the female mice taken post-exercise. Further histological analysis showed that this signal likely comes from the presence of muscle fibres from the panniculus carnosus muscle inside the tumours. Also, we found no differences in the positivity for the proliferation marker KI67 in the control and exercise C26 carcinomas.

**Conclusion:**

A bout of exercise did not systematically affect gene expression or proliferation in C26 carcinomas in immunocompetent BALB/c mice.

## Introduction

Exercise is an effective treatment for many diseases including cancer (Pedersen and Saltin 2015). In 2018, a review by Christensen and colleagues summarised research of 700 exercise intervention trials with more than 50,000 cancer patients (Christensen et al. 2018). The research covered demonstrated how exercise affects disease progression, physical and psychosocial well-being, interaction with anti-cancer therapies and specific cancer outcomes, such as reduced recurrence and improved survival. Because exercise has mainly beneficial effects for cancer patients, some associations such as the Clinical Oncology Society of Australia COSA now recommend exercise as an adjunct treatment for cancer patients where exercise is not contraindicated (COSA 2017).

Several studies indicate that exercise training can also reduce the growth of cancers and/or proliferation of cancer cells. For example, voluntary wheel running reduces the growth of melanomas, lung cancers, colon cancers, breast cancers and hepatocarcinomas in immune-competent mice by 31–67% and of human breast cancers and head and neck cancers in immune-incompetent mice by 26–54% (Hojman et al. 2018). Moreover, incubating human cancer cell lines with exercise-conditioned blood sera generally reduces proliferation of human cancer cell lines cultured in vitro, when compared to incubation with control blood sera (Metcalfe et al. 2021). Observational studies show that higher levels of physical activity are associated with higher survival rates in 2,987 women with breast cancer (Holmes et al. 2005), 573 women with colorectal cancer (Meyerhardt et al. 2006) and 2,705 men with prostate cancer (Kenfield et al. 2011). In a randomised clinical trial, Courneya et al. investigated the effect of endurance or resistance training during chemotherapy in 242 women with breast cancer. The study suggests a better disease-free survival in the pooled exercise than in the control group (Courneya et al. 2014). A recent systematic review and meta-analysis of eight randomised control trials concluded that exercise significantly reduced the risk of recurrence (risk reduction 0.52) and mortality (risk reduction 0.76) in cancer patients and survivors (Morishita et al. 2020). Together, this suggests that exercise does not only improve the general condition and well-being of cancer patients, but can also affect hallmarks of cancer and improve outcomes such as survival and recurrence.

Some studies were carried out to elucidate the mechanisms by which exercise affects cancer hallmarks (Hanahan and Weinberg 2011), cancer growth as well as cancer outcomes such as recurrence and survival (Hojman et al. 2018). Generally, exercise alters the concentrations of many blood molecules including hormones (e.g. catecholamines), metabolites (e.g. lactate, glutamine (Pedersen et al. 2020)) nucleotides, RNAs (Contrepois et al. 2020) and proteins (e.g. exerkines (i.e. signalling molecules released in response to exercise) such as FGF21) (Contrepois et al. 2020; Schranner et al. 2020) and of blood cells, especially immune cells. These molecules can either be free or in extracellular vehicles (Whitham et al. 2018) or in blood cells. Exercise-conditioned blood will also perfuse a tumour, and the cancer cells or other cells within the tumour environment can respond to the exercise-modulated factors if they express the matching receptor (e.g., adrenoreceptors for catecholamines) or if the exercise-altered molecules interfere in another way with the signalling, metabolism or immune function of cells within the tumour environment.

In relation to this, one major focus of mechanistic studies were the catecholamines adrenaline (American English “epinephrine”) and noradrenaline (American English “norepinephrine”). These hormones increase during exercise and in response to stress (Tank and Wong 2015; Wackerhage et al. 2022). Whilst exercise studies suggest that exercise-induced catecholamines promote the migration of natural killer cells into a tumour (Pedersen et al. 2020) or activate tumour-inhibiting Hippo signalling (Dethlefsen et al. 2017), other studies in the context of psychosocial stress suggest that catecholamines promote, e.g. pancreatic adenocarcinoma (Renz et al. 2018). We have recently reviewed the variable effects of exercise in cancer, terming it the “cancer catecholamine conundrum” (Wackerhage et al. 2022).

Given that exercise alters concentrations of blood-based regulators and given that cancer cells express receptors for many of these factors (Ghandi et al. 2019), cells within a tumour should alter their signal transduction and gene expression after a bout of exercise. For example, rises of adrenaline, noradrenaline (Wackerhage et al. 2022) and interleukin-6 (Orange et al. 2023) during exercise will alter cAMP-PKA or Jak/Stat3 signalling, respectively. Whilst changes of gene expression are a key cause for altered cancer cell behaviour (e.g. proliferation is reliant on the expression of genes such as *TP53* or *PCNA*), not all changes of cell behaviour are caused by changes of gene expression. For example, the glucose uptake response to insulin is mediated primarily by protein phosphorylation and not changes in gene expression (Wasserman 2022).

The aim of this project was to investigate whether a bout of exercise alters gene expression in a tumour. Specifically we sought to answer the following three research questions:

1. Do C26 mouse colon cancers express receptors for exercise hormones and exerkines?
2. How does one bout of running exercise alter gene expression in a C26 mouse colon cancer in immunocompetent mice (Geremia et al. 2022)?
3. Does one bout of running exercise affect proliferation in a C26 mouse colon cancer?

## Materials and methods

This study was not registered prospectively as it is not a human clinical trial.

### C26 colon cancer model

We used a C26 colon carcinoma in BALB/c mice as a cancer model. C26 cells were cultured in high-glucose DMEM (# 41,966 Gibco) supplemented with 10% of foetal bovine serum (Life Technologies) and Pen/Strep solution (penicillin 100 U/ml and streptomycin 0.1 mg/mL, Gibco). Cell culture was maintained at 37° C with a humidified atmosphere of 5% CO_2_. Low-passage cell lines were used. To initiate a tumour, we injected a C26 cell suspension subcutaneously, dorsally into 3-month-old mice. Tumour-bearing mice received 100,000 C26 cells in PBS and were divided into two different groups: rest (control, *n* = 3 females, 2 males) and exercise (*n* = 3 females, 3 males). When C26 mice had lost 10% of their body weight when compared to the day of inoculation (D0), they performed one bout of exercise in the morning and immediately after that, we collected the C26 tumour and a serum samples. All mice lost weight at a similar rate, as the C26 model is quite reproducible when inoculating cells from the same preparation. The treadmill exercise was modified from Solagna et al., and assured a sustained run which also tumour-bearing animals were able to sustain for the whole duration (65 min). The rested control group was sacrificed at the same time point. The run protocol used for the single bout of exercise is described in Table 1. Blood serum samples were collected as follow: mice were anaesthetised with isoflurane and blood was collect retro-orbitally with a glass pipet. Experimental protocols were reviewed and approved by the Ethical Committee, University of Padova. "Principles of laboratory animal care" (NIH publication No. 85–23, revised 1985) were followed, as well as Italian national law (the animal experiments were performed in Italy).

**Table 1:** Exercise protocol (800 m in 65 min)

Time	Velocity
10 min	16 cm/s
10 min	18 cm/s
10 min	20 cm/s
10 min	22 cm/s
10 min	23 cm/s
10 min	25 cm/s
5 min	26 cm/s

### Library preparation and sequencing

To identify transcriptional changes after one bout of exercise, approximately 100 mg of cancer tissue was cutoff on dry ice, transferred to TRIzol (Invitrogen), homogenised three times at 4,000 rpm for 10 s two times using a high-throughput tissue homogenizer (Precellys 24, Bertin Technologies). The homogenates were then centrifuged at 14,000*g* for 1 min at 4 °C to remove tissue debris. Total RNA was isolated from the Trizol homogenates using the Direct-zol™ RNA MiniPrep Kit (Zymo Research) and RNA integrity was validated using BioAnalyzer (Agilent). cDNA sequencing libraries were generated with the CORALL mRNA-Seq V2 (Lexogen) according to the manufacturers’ protocol. All libraries were sequenced on the same run using a NEXTSEQ™1000 device (Illumina) with a read length of 50 bp and a sequencing depth of approximately 20 million reads per sample.

### Bioinformatic analysis

Sequencing reads were pseudo-aligned to the mouse reference genome (version GRCm39.106) with kallisto (version 0.48). Post-processing was performed in R/bioconductor (version 4.4.2) using default parameters if not indicated otherwise. Differential gene expression analysis was performed with DEseq2 (version 1.36.0). An adjusted *p* value (FDR) of less than 0.1 was used to classify significantly changed expression.

### KI67 proliferation stain

Tissue samples were fixed in 10% neutral-buffered formalin for a minimum of 48 h and processed for histology and then stained with haematoxylin and eosin (H&E) staining using standard protocols. Immunohistochemistry (IHC) was performed using a Bond RXm automated system (Leica, all reagents from Leica, Wetzlar, Germany). A primary antibody against KI67 (Abcam, ab16667, clone SP6) was applied. Primary antibody binding was detected with a polymer refine detection kit (DS9800, Leica) without a post-primary antibody and visualised by incubation with 3,3’-diaminobenzidine (DAB). Slides were counterstained with haematoxylin, put in distilled water, dehydrated manually and coverslipped on an automated coverslipper (CTM6, Thermo Scientific, Waltham, USA) using CytosealTM XYL mounting solution (8312–4, Thermo Scientific).

### KI67 manual and automatic counting

First, we quantified the percentage of KI67-positive cells blinded and manually in subjectively identified KI67 hotspots (4 fields of view at a 40 × magnification) using ImageJ version v53w. A positive count was any brown stain in the nucleus above the background; a negative stain was scored when the invasive cancer cell showed only a blue counterstained nucleus. In addition to the manual KI67-hotspot analysis, we used QuPath 0.3.2 (Open Software for Bioimage Analysis (Robertson et al. 2020) to analyse scanned IHC slides and to assess the KI67 percentage of positive cells. Before analysing pictures for positive cells, smoothing filter was applied. The smoothing command reduces noisiness between cells. To count and detect KI67-positive cells, we used ''positive cell detection'' in the image with the following settings: detection image, optical density sum; requested pixel size, 0.5 µm; background radius, 8 µm; median filter radius, 0 µm; sigma, 1.5 µm; minimum cell area, 10 µm^2^; maximum cell area, 400 µm^2^; threshold, 0.1; maximum background intensity, 2. The nucleus DAB optical density mean of DAB-positive cells (the average of brown staining within the nucleus) was detected using positive cell detection with thresholds of 1 + , 2 + and 3 + to detect a variety of positive expressed cells in the QuPath. The same protocol (estimate stain vectors, annotation of area of interest, smoothing, and positive cell detection) was used for every slide analysis.

### Statistical analysis

Statistical analysis of the KI67 stains was performed with Jasp Team (Version 0.16.1.0). Since data were not normally distributed as judged by a Shapiro–Wilk test, we performed a Mann–Whitney U test to test for differences in between the rested and exercised animals.

## Results

### C26 colon carcinomas express receptors for exercise-related hormones

**Fig. 1:**
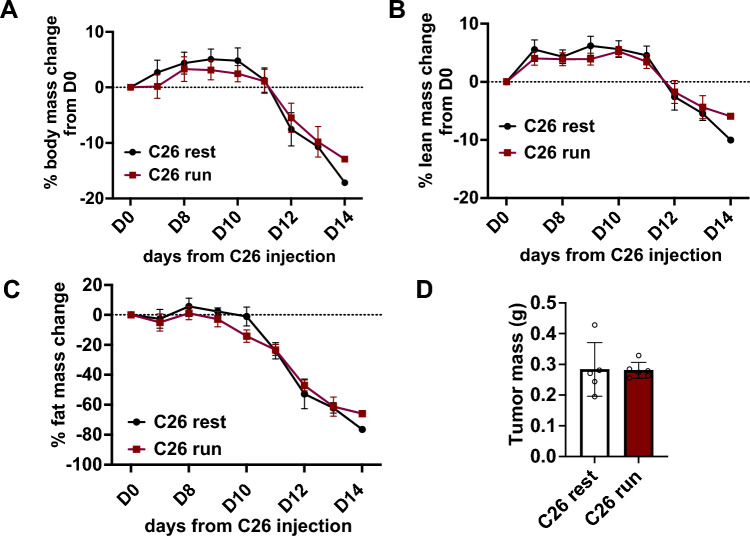
**A** Change of body weight in mice inoculated with C26 cells over time. Change of lean **B** and fat **C** mass over time as determined by echo-MRI, **D** tumour weight at the time of exercise

In this study, we investigated whether one bout of exercise would systematically change gene expression in a C26 colon carcinoma in 3-month-old Balb/c mice. As can be seen in Fig. 1, mice lost a significant amount of both lean and fat mass at 14 days after inoculation, without any difference in tumour mass between the two groups.

**Fig. 2:**
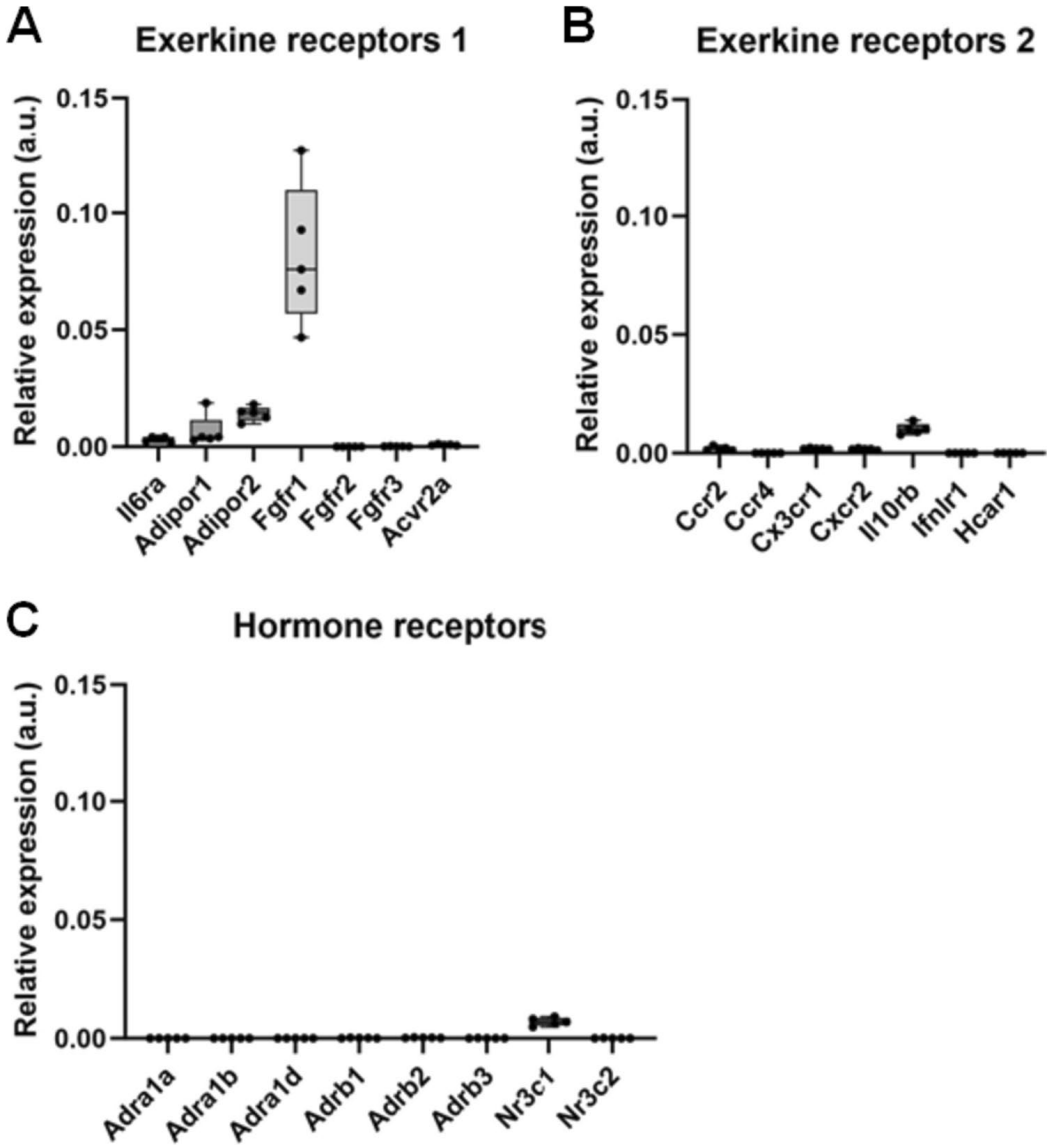
C26 mouse colon tumours taken from the rested mice express some receptors for exerkines **A**, **B** and hormones **C**. The expression values in transcripts per million for each receptor were normalised by dividing them by the expression value for glyceraldehyde-3-phosphate dehydrogenase (*Gapdh*)

Cancer cells can only respond to an exercise-regulated hormone or to other regulatory factors if they express the receptor or other target for that factor. To find out whether C26 cells express receptors for known regulatory factors that change their concentration during exercise, we measured their gene expression in the C26 tumours from the resting mice. This data is shown in Fig. 2. The receptors shown include receptors for catecholamines (the receptor gene symbols are: *Adra1a/b/d, Adrb2, Adrb3*), cortisol (*Nr3c1/3*), and the exerkines interleukin-6 (*Il6ra*) adiponectin (*Adipor1/2*), angiopoietin-1 (*Tie1*), Bdnf, Fgf21 (*Fgfr1/2/3*), myostatin (*Acvr2a*), Ccl2 (*Ccr2/4*), Cx3cl1 (*Cx3cr1*), Cxcl2 (*Cxcr2*), Ifnl3 (*Ifnlr1*) and lactate (*Hcar1*). This suggests that C26 tumours express some receptors for some exercise-modulated hormones and exerkines such as Fgfr1.

### How does one bout of running exercise alter gene expression in a C26 mouse colon cancer in immunocompetent mice?

**Fig. 3:**
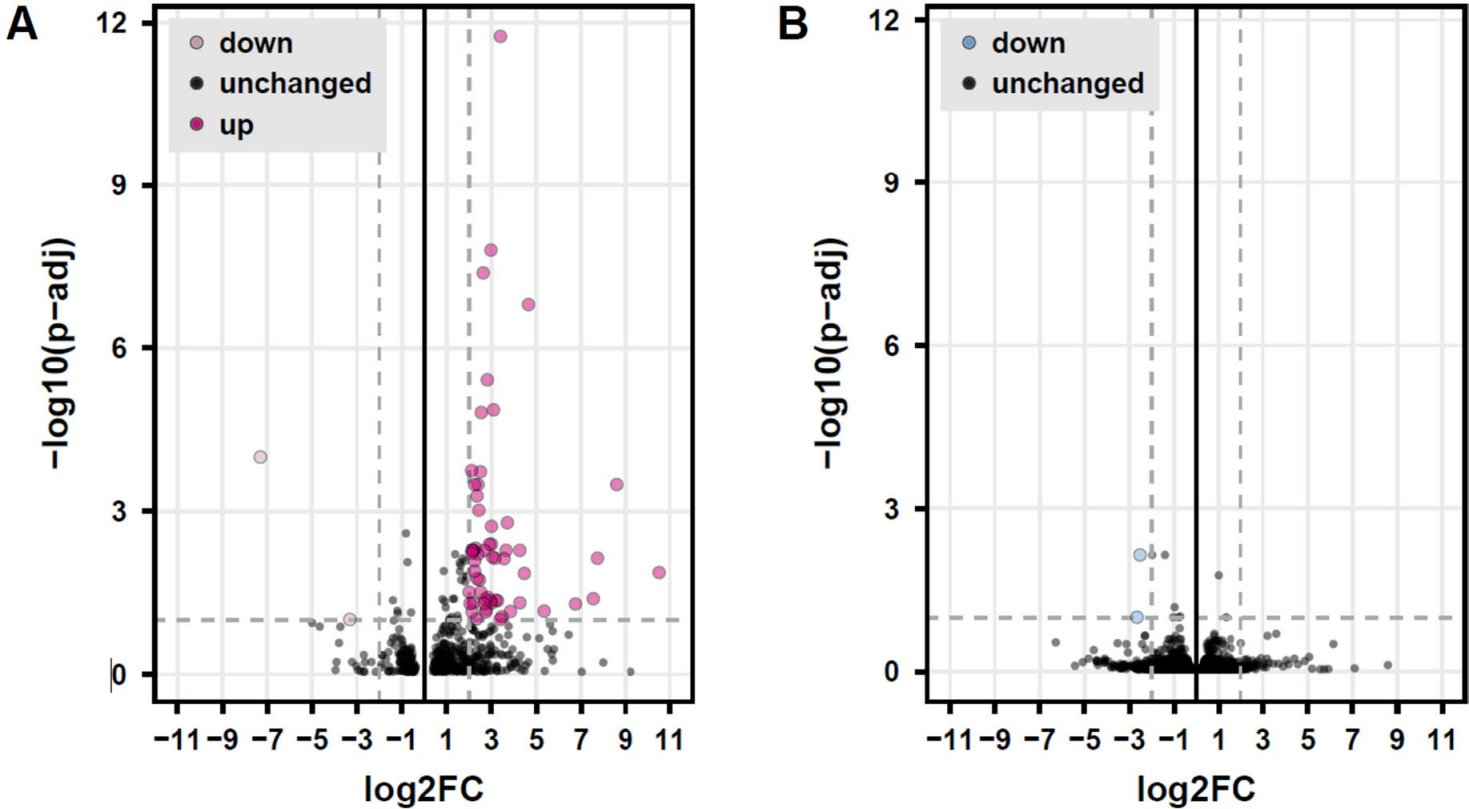
Volcano plot visualising the effect of one bout of running on gene expression in a C26 mouse colon tumour in female (**A**; three samples rest, three samples post-run) and male (**B**; two samples rest, three samples post-run) mice

Given that that the C26 mouse colon cancer cells express receptors for some regulatory factors that typically change their concentration in response to exercise, we hypothesised that this would trigger systematic changes in C26 tumour gene expression. We found, however, that exercise did not significantly change the expression of the tumours of the mice that had exercised versus the tumours of the mice that rested. When analysing the data of the female and male mice separately, we identified some significantly upregulated and downregulated genes. An enrichment analysis with Toppgene (Chen et al. 2009) revealed that over 10 of the 17 significantly upregulated genes in the tumours of the female mice were skeletal muscle genes such as *Acta1, Actn3, Myh2, Myh4* and *Des* (Fig. 3).

**Fig. 4:**
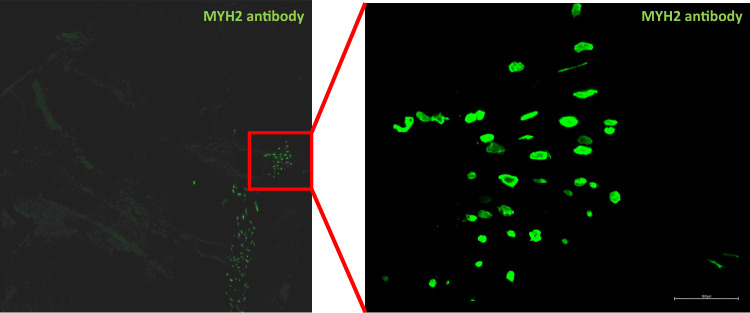
Staining for myosin heavy chain 2A (encoded by *Myh2*) reveals some muscle fibres inside C26 tumours. Most likely these fibres correspond to fibres from the panniculus carnosus muscle

Histological analysis of the C26 tumours showed that these increases in muscle transcripts are due to the presence of muscle fibres inside the tumour. As these tumours are inoculated underneath the skin, they can grow whilst incorporating most likely part of the panniculus carnosus muscle, a thin layer of skeletal muscle localised within the subcutaneous layer of the skin (Naldaiz-Gastesi et al. 2018). This very small layer of muscle fibres, hardly present in humans, expresses fast myosin heavy chain as can be seen in Fig. 4. We find that the amount of these sparse muscle fibres, whilst present in more than 80% of tumours examined, is not consistent between tumours. The observed difference in muscle transcripts between different groups can, therefore, potentially be attributed to different amounts of muscle fibres inside the tumour, not by changes of expression from the tumour cells themselves.

Overall, the lack of an effect of exercise on cancer hallmark-related C26 tumour gene expression (e.g. proliferation genes) is surprising because Hojman et al. reported that voluntary wheel running reduces the size of mouse C26 colon cancers in immunocompetent mice by 31% (Hojman et al. 2018).

### Does one bout of running exercise affect proliferation in a C26 mouse colon cancer?

**Fig. 5:**
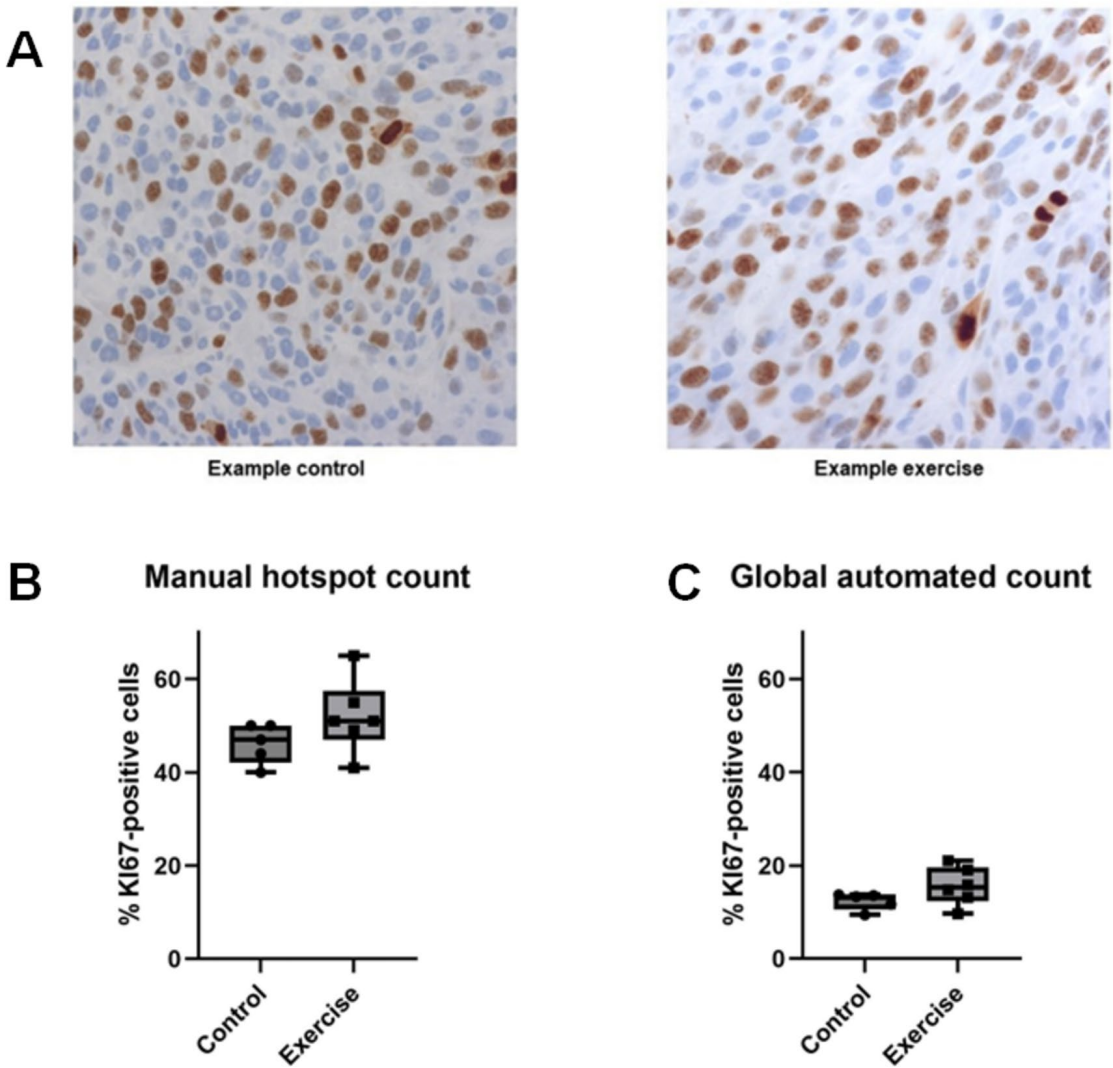
**A** Example of a KI67-rich hotspot stain of a C26 tumour from rested control mice (left) and exercised mice (right). Manual **B** and automated **C** count of KI67-positive cells in KI67 cell-rich hotspots or the whole area of C26 tumours in mice at rest or mice that had exercised. There was no statistical difference between the resting control and exercise mice

Based on the previous observation that voluntary running reduces the growth of C26 colon cancers by a third in immunocompetent mice, we studied whether exercise altered the percentage of KI67-positive cells in the C26 colon cancers. We found by blinded, manual counting of KI67 hotspots that the C26 colon cancers of the five resting control mice had 46 ± 4% KI67-positive cells in KI67 hotspots (Fig. 5). In contrast, the C26 colon cancers of the exercised mice had 52 ± 8% KI67-positive cells in KI67 hotspots. In addition to the manual analysis, we automatically determined the percentage of KI67-positive cells in whole tumours. This revealed 12 ± 2% KI67-positive cells in the whole C26 tumours of the mice that had rested and 16 ± 4% of KI67-positive cells in the C26 tumours of the mice that had exercised. These differences were not statistically significant.

Together, these data suggest that a single bout of incremental running does not affect gene expression or the number of KI67 positive cells in a C26 carcinoma in mice.

## Discussion

The main finding of this study is that a single bout of exercise has no effect on the gene expression and proliferation of C26 colon cancers in immunocompetent mice. This is surprising because the C26 colon cancers expressed some receptors for exercise-modulated blood regulators and because an earlier report suggested that voluntary wheel running was reducing the growth of these cancers by 31% (Hojman et al. 2018).

The key question is how to explain the lack of effect of running on intra-tumour gene expression and proliferation? One possible explanation might be thatthe 65 min exercise bout was too short to alter gene expression. However, this seems unlikely. For example, a meta-analysis of human muscle biopsy studies shows that a bout of endurance or resistance exercise systematically alters the expression of hundreds of genes directly after exercise (Pillon et al. 2020). A caveat is that muscle is directly exposed to signal transduction and gene expression-changing stimuli such as AMP, Ca^2+^ or glycogen and does not rely on exercise-modulated blood factors like a tumour. However, white adipose tissue gene expression responds to 120 min of swimming in mice (Shen et al. 2016). This suggests tissues that are regulated by exercise-regulated circulating factors such as catecholamines can experience  a changed gene expression directly after a bout of exercise.

The finding that gene expression was unchanged does not mean that there were no effects on the tumour as exercise can alter tumour behaviour without altering gene expression. One example would be exercise-induced changes of concentrations of substrates such as glucose or amino acids that tumours need for their anabolism. However, most hallmarks of cancer will be accompanied by changes in gene expression. For example, proliferation requires the expression of genes that manage mitosis, angiogenesis requires the expression of angiogenesis regulators and of genes that encode blood vessel proteins and inflammation will be linked to an increased inflammation-associated expression signature.

An unexpected observation was the fact that there is some muscle tissue present especially inside the female C26 tumours. As these muscle fibres express a mature form of MYH, this cannot be regenerating fibres because of the presence of the tumour, as regeneration requires multiple weeks before it expresses mature MYH and reaches normal dimensions and no central nuclei were observed. As these muscle fibres are localised sparse throughout the tumour, it fits well with the small layer of muscle fibres expressed in the superficial layer of the skin, where inoculation occurs. This also means that one must be careful in analysing whole tumour analyses, as they can contain multiple, somewhat unexpected, cell types. Finally, we do not know why muscle genes were mainly expressed in the C26 tumours of female mice. To us the likely explanation is normal variation.

This study has limitations. First, we only investigated in one mouse cancer model (i.e. C26 carcinoma), in a proof-of-principle experiment in immunocompetent mice, whether one bout of exercise systematically affects gene expression within a tumour at the end of 65 min of exercise. Future studies should investigate more variables (e.g. gene expression and protein abundance) and investigate more time points (e.g. no exercise, direct after exercise, 1 h post-exercise, 3 h post-exercise). Second, we compared the tumours of five mice at rest (three males, two females) with those of six mice post-exercise (three males, three females) and so the numbers of a sex-specific analysis, e.g. of gene expression, are small. A third limitation is that we have not verified if e.g. hormones, exercise or metabolites change during exercise. However, exercise changes the concentration of thousands of molecules in blood which is well documented (Contrepois et al. 2020). The fourth limitation is that changes in gene expression do not always mean similar changes of protein concentrations. For example, the data in Fig. 2 suggest that C26 tumours express receptors for exercise-modulated circulating regulators but it is possible that some of these mRNAs are not translated into the receptor protein.

## Conclusion

This study shows in a C26 colon carcinoma mouse model in immunocompromised mice that at least some cancers do not respond with a systematic change of gene expression to a single bout of incremental running exercise even though C26 carcinomas express some receptors for exercise-regulated factors. Future studies should investigate different types of exercise, time points post-exercise and different cancers to better understand the previously reported reductions in tumour growth after exercise training (Hojman et al. 2018).

## Data Availability

The data that support the findings of this study are available from the corresponding author, Henning Wackerhage, upon reasonable request.
